# Intracellular microbes empower cancer metastasis

**DOI:** 10.1093/lifemedi/lnac009

**Published:** 2022-06-28

**Authors:** Aikun Fu, Bingqing Yao, Tingting Dong, Shang Cai

**Affiliations:** Westlake Laboratory of Life Sciences and Biomedicine, Hangzhou 310024, China; School of Life Sciences, Westlake University, Hangzhou 310024, China; Westlake Laboratory of Life Sciences and Biomedicine, Hangzhou 310024, China; School of Life Sciences, Westlake University, Hangzhou 310024, China; Westlake Laboratory of Life Sciences and Biomedicine, Hangzhou 310024, China; School of Life Sciences, Westlake University, Hangzhou 310024, China; Westlake Laboratory of Life Sciences and Biomedicine, Hangzhou 310024, China; School of Life Sciences, Westlake University, Hangzhou 310024, China

Cancer is a dynamic ecosystem that is constituted by a number of biotic components such as cancer-associated fibroblasts, endothelia, and immune cells, as well as abiotic components such as pH and oxygen level. Cancer cells’ survival and growth are largely dependent on the micro-environmental niche that provides nutrients, signaling molecules, and metabolites to sustain and determine the cancer progression trajectory. Targeting the micro-environment components as therapeutic strategy has emerged as a promising cancer treatment in research and clinics. A hundred years ago, William Coley utilized bacteria to treat cancer patients and achieved promising cancer regression. He hypothesized that tumors may all have a microbial origin [1]. In recent years, intratumoral microbiota, as a novel tumor component, has been gradually revealed due to the rapid development of next-generation sequencing. These bacteria have an intriguing localization inside cancer cells in various tumor types [2–4], but the biological meaning remains unclear. As bacteria have the potential to crosstalk with the host cells and the immune system by metabolites and signaling molecules [5], understanding how intratumoral microbiota is involved in tumor progression is a pressing scientific question to address with potential clinical implications.

Recently, this question has been addressed in a spontaneous murine breast tumor model MMTV-PyMT, in which primary tumor is frequently accompanied with lung metastases [6]. The study started to validate whether this tumor model can represent the features of the reported human breast cancer. As the long-standing challenge to characterize tissue-resident microbiota is the environmental contamination and host genome contamination [7], the study optimized the quantification and profiling method to be able to faithfully detect 10^3^ bacteria/g tissue. The optimized method revealed 10^5^ bacteria/g tissue in PyMT tumor, similar to what was quantified in human breast tumor tissues [3]. Interestingly, the intratumoral microbiota was enriched mainly in gram-positive, facultative anaerobic Firmicutes such as *Enterococcus*, *Staphylococcus*, *Streptococcus*, and *Lactobacillus*, all of which can be found in human breast tumor microbial taxa [3, 6]. Furthermore, through Electron Microscopy analysis and treatment of antibiotics targeting extracellular/intracellular bacteria, the intracellular location of tumor resident microbiota in PyMT was demonstrated, which represents the intracellular feature of bacteria (e.g. escaping the endosome restriction) in human tumor tissue, making it an ideal tumor model to interrogate bacteria’s role in tumor progression.

The crux of intratumoral microbiota study lies in the functional role of these microbes [7]. As the abundance of intratumoral microbiota is far lower than gut microbiota, the way that intratumoral microbiota exert their functions is supposed to be distinct. Indeed, eradicating the intratumoral microbiota alone with minimal perturbation of gut microbiota by distinct antibiotic combinations and administration routes considerably reduced lung metastasis, leaving the primary tumors unaffected [6]. The antibiotic treatment of bacteria-bearing tumors on germ-free mice reinforced the assertion, indicating that intratumoral bacteria have unique and independent roles from gut microbiota. This phenotype is in accordance with the recent finding that *Fusobacterium nucleatum* colonizes to breast cancer through Fap2-Gal-GalNAc interaction, and inoculation of *F. nucleatum* in breast tumor accelerates metastasis [8].

As a microbial community, the intratumoral bacteria could function coordinately as a group, or separately as individual species. The latter was verified by introducing each isolated bacteria strains back into the tumor [6]. Except for *Enterococcus faecalis*, all other isolated bacteria strains including *S. xylosus*, *S. cuniculi*, and *L. animalis* were able to efficiently invade into the cytoplasm of PyMT tumor cells *in vitro*, and promote tumor cell lung colonization and tumor macro-metastasis development *in vivo* after intratumoral injection of these strains. Most intriguingly, for a non-metastatic tumor type MMTV-Wnt, which contained very low amount of intratumoral bacteria, the intratumoral administration of these bacteria strains at the physiological loads drastically increased the lung metastasis. This phenotype implies that although tumor cells’ behavior is largely controlled by their genetic configuration, the intratumoral microbiota could also be an essential etiological factor that determine some aspects of cancer progression. In the clinics, the characterization of intratumoral microbiota may therefore need to be carefully considered for precision medicine.

If intratumoral bacteria promote tumor metastasis, then do bacteria enhance the host cell’s metastatic ability, or the neighboring cells? In theory, if bacteria promote host cell metastasis, the microbial presence in the lung mets and the circulating tumor cells should be evident. Indeed, the microbiota analysis of normal breast tissue, primary breast tumor, small lung mets, and macro lung mets clearly showed the persisting bacteria strains from primary site to metastasis site [6]. Furthermore, the presence of bacteria in the clustered circulating tumor cells was demonstrated by FISH analysis. And this conclusion was further nailed down by a genetic tracing experiment showing that the genetically labeled bacteria strain can only colonize in the lung tissue by hijacking the tumor cells, consistent with the previous report in colon cancer that bacteria persisted in tumors along with passages and in metastasis [9].

**Figure 1.**
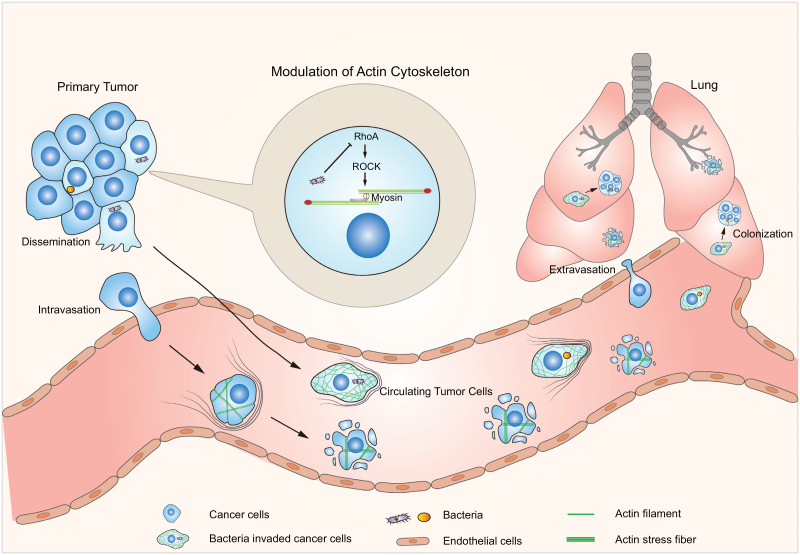
**Schematic diagram illustrating the role of intracellular microbiota in cancer metastasis.** Various strains of bacteria were identified in murine breast tumor model MMTV-PyMT with a physiological abundance around 10^5^ bacteria/g tissue. These bacteria do not play appreciable roles in primary tumor growth, but they can travel the circulation system together with the metastatic tumor cells. When metastasizing, intracellular bacteria play crucial roles in helping the tumor cells to resist the fluid shear stress, which eventually triggers cell contraction and kills the vast majority of circulating tumor cells. Intracellular bacteria promote cancer cell survival by their ability to repress the activation of RhoA-ROCK-Myosin light chain signaling pathway to reorganize actin cytoskeleton, favoring cell’s attachment and spreading. This facilitates cancer cell’s metastatic colonization which ultimately develops into macro-metastases.

Metastasis is the major cause of breast cancer patient mortality. It is a multistep biological process involving dissemination, intravasation, extravasation, and colonization. Major factors mediating metastasis have been attributed to epithelial–mesenchymal transition and immune surveillance [10]. Through single-cell sequencing analysis of the bacteria invaded PyMT tumor cells, the study revealed that the intracellular bacteria played a dominant role in modulating cancer cells’ fluid shear stress response. An *in vitro* circuit setup mimicking blood flow in the vessel tested the stress response ability and showed that bacteria invaded tumor cells had considerably improved survival ability against fluid stress damage. Various bacteria strains, except *Enterococcus*, did this by modulating a RhoA-ROCK signaling pathway, which in general triggers cytoskeleton contraction with subsequent cell death [6] (Fig. 1).

The new finding of the protective role of intratumoral bacteria for cancer cells against mechanical stress during metastasis is an important discovery for our understanding of cancer progression. It nicely complements to the current cancer metastasis theory, which explicitly explains why cancer cells migrate, disseminate, and evade immune surveillance, but remains obscure in the underlying law governing stress response in early colonization. This finding echoes William Coley’s suspicion a hundred years ago that ‘all varieties of malignant tumors are of extrinsic or microbic origin’ [1], and entails the further in-depth investigation of how intratumoral bacteria at its physiological abundance integrates into various processes of cancer progression. Of note, the discovered role of intratumoral microbiota in tumor progression could be more comprehensive than just breast cancer, as there was evidence in colon cancer that bacteria persisted during cancer metastasis [9]. This study also implicates that, besides the well-known genetic factors and cellular communications impinging on the tumor progression, the intratumor microbiota could be another component equally important in determining cancer patient prognosis; therefore, targeting the intratumoral microbiota may have the potential to be developed as a novel therapeutic approach. The presence and involvement of microbes in cancer might just be the surface of the truth, and the underlying laws governing the composition, distribution, abundance, invasion, etc. might really embody the fundamental dogma of cancer progression that allows us to understand better the essence of cancer.
